# Supplementary material for: Antimicrobial Activity of Different Artemisia Essential Oil Formulations
Source: Molecules. 2020 May 21;25(10):2390. doi: 10.3390/molecules25102390 (PMC7287661; doi:10.3390/molecules25102390)
Supplement: Supplementary file 1 [file molecules-25-02390-s001.pdf]

Antimicrobial Activity of Different *Artemisia* Essential Oil Formulations

Sourav Das [1,2,†], Barbara Vörös-Horváth [3,†], Tímea Bencsik [4], Giuseppe Micalizzi [5], Luigi Mondello [5,6,7], Györgyi Horváth [4], Tamás Kőszegi [1,2,*] and Aleksandar Széchenyi [3,*]

[1] Department of Laboratory Medicine, University of Pécs, Medical School, 7624 Pécs, Ifjúság u. 13., Hungary; pharma.souravdas@gmail.com

[2] János Szentágothai Research Center, University of Pécs, 7624 Pécs, Ifjúság u. 20., Hungary

[3] Institute of Pharmaceutical Technology and Biopharmacy, University of Pécs, Faculty of Pharmacy, 7624 Pécs, Rókus u. 2., Hungary; barbara.horvath@aok.pte.hu

[4] Department of Pharmacognosy, University of Pécs, Faculty of Pharmacy, 7624 Pécs, Rókus u. 2., Hungary; timea.bencsik@aok.pte.hu (T.B.); horvath.gyorgyi@gytk.pte.hu (G.H.)

[5] Department of Chemical, Biological, Pharmaceutical and Environmental Sciences, University of Messina, 98168, Messina, Italy; giumicalizzi@unime.it (G.M.); lmondello@unime.it (L.M.)

[6] Chromaleont s.r.l., c/o Department of Chemical, Biological, Pharmaceutical and Environmental Sciences, University of Messina, 98168, Messina, Italy

[7] Unit of Food Science and Nutrition, Department of Medicine, University Campus Bio-Medico of Rome, 00128, Rome, Italy

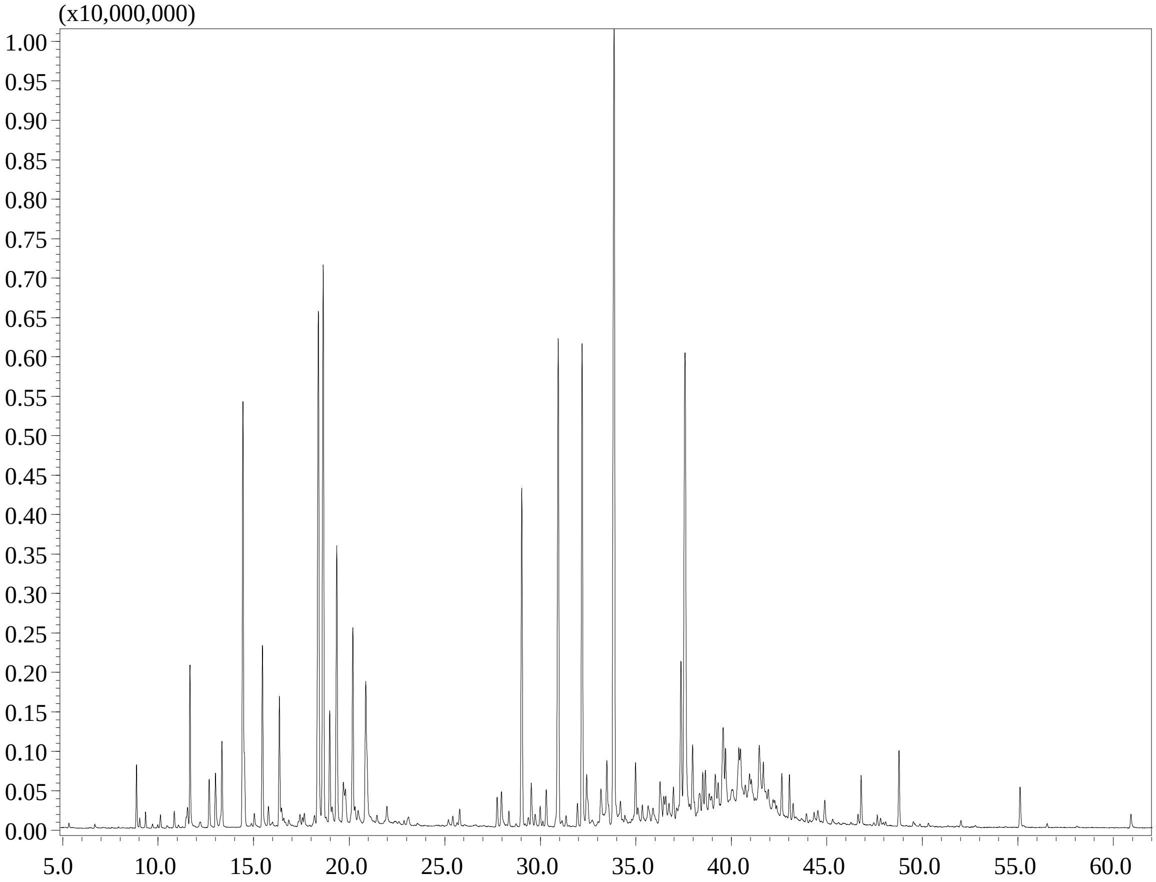

Supplementary Figure S1. GC-MS chromatogram of the *Artemisia annua* essential oil on SLB-5ms column.

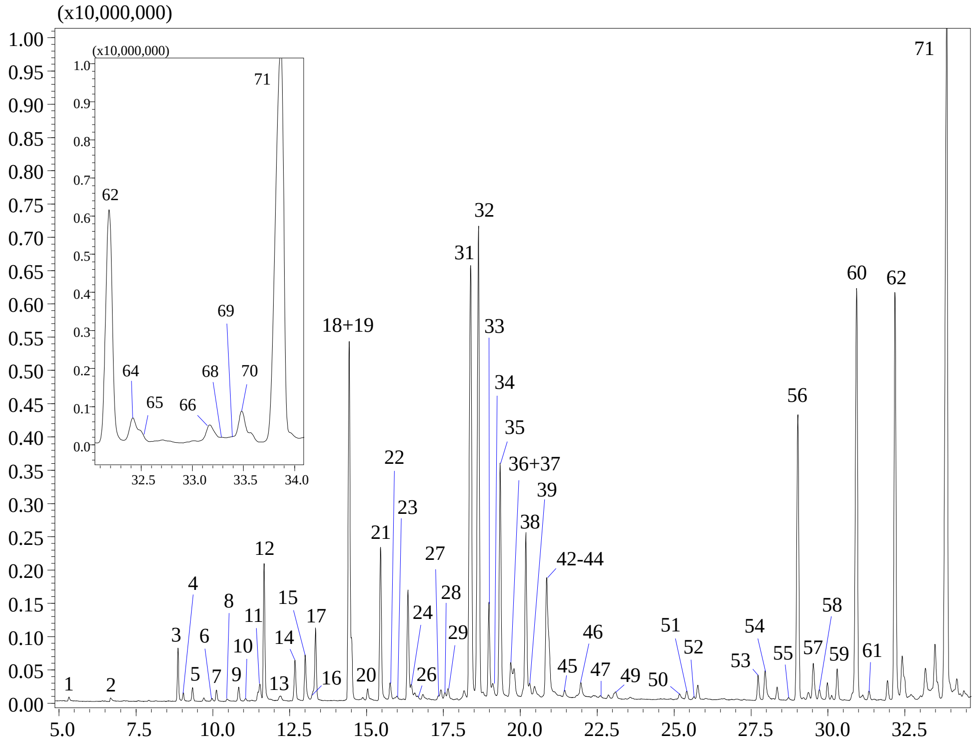

Supplementary Figure S2. Expansion (5-35min) of the GC-MS chromatogram of the *Artemisia annua* essential oil on SLB-5ms column.

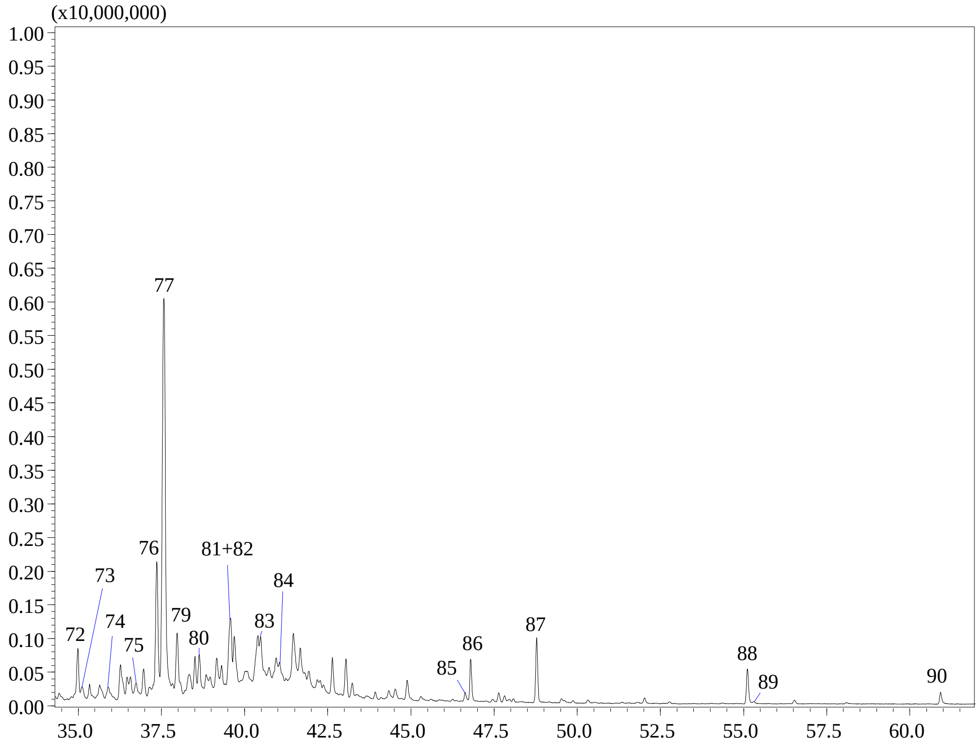

Supplementary Figure S3. Expansion (35-63min) of the GC-MS chromatogram of the *Artemisia annua* essential oil on SLB-5ms column.

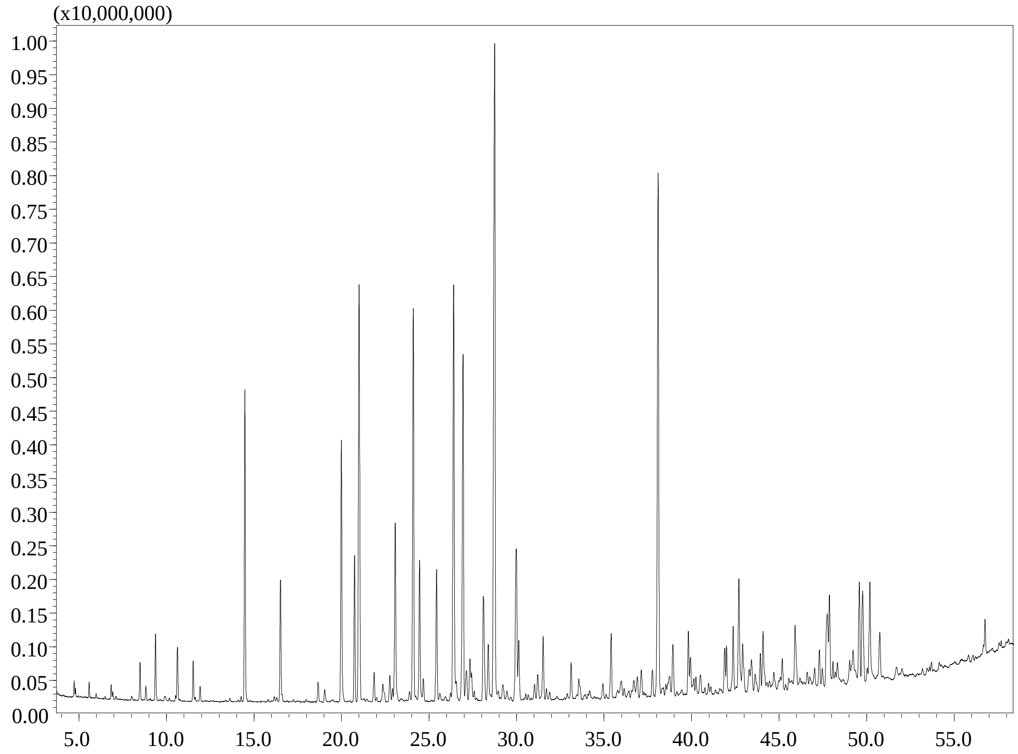

Supplementary Figure S4. GC-MS chromatogram of the *Artemisia annua* essential oil on Supelcowax-10 column.

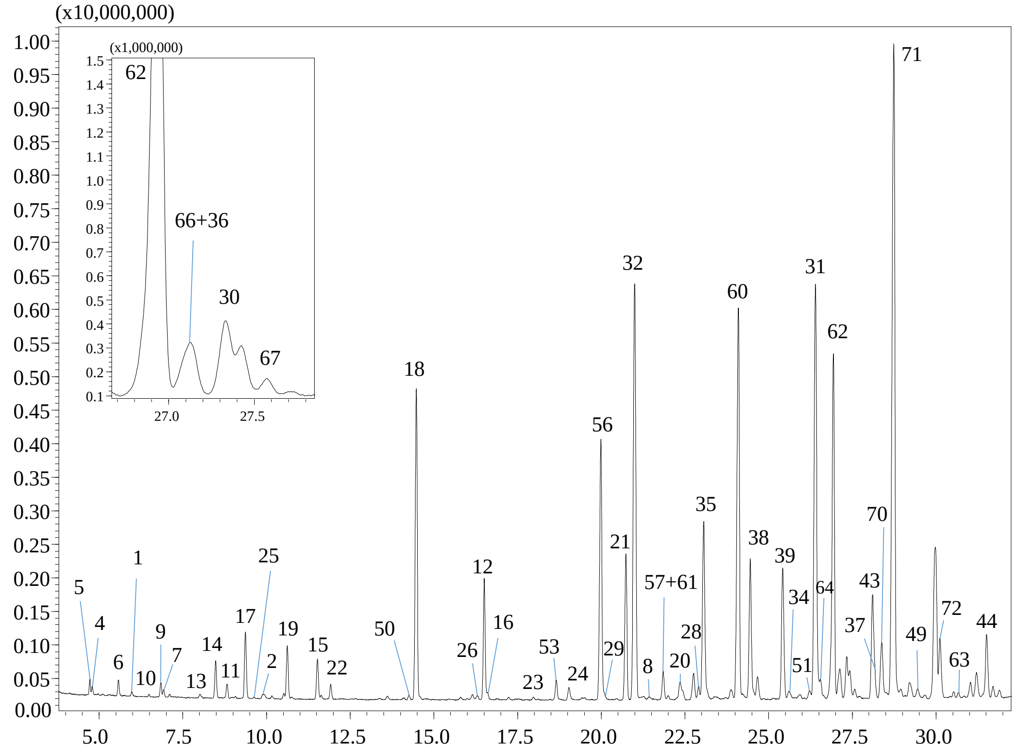

Supplementary Figure S5. Expansion (4-32min) of GCMS chromatogram of the *Artemisia annua* essential oil on Supelcowax-10 column.

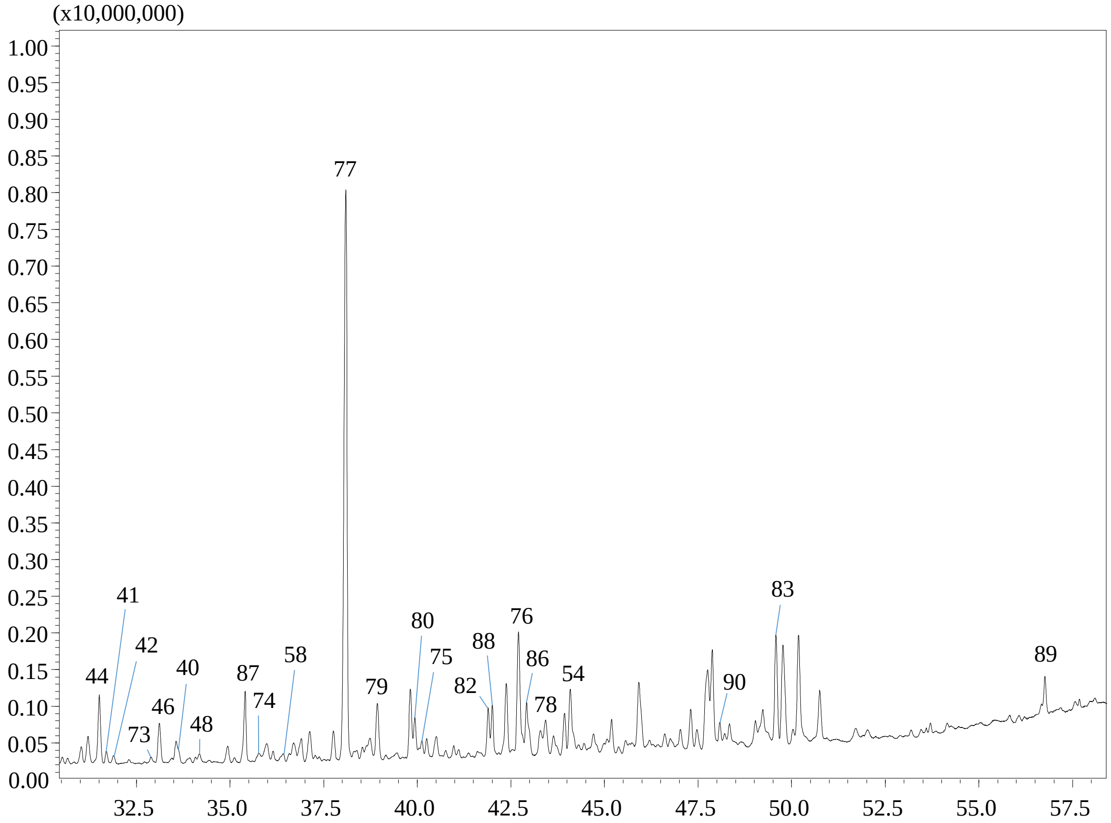

Supplementary Figure S6. Expansion (32-58min) of the GCMS chromatogram of the *Artemisia annua* essential oil on Supelcowax-10 column.

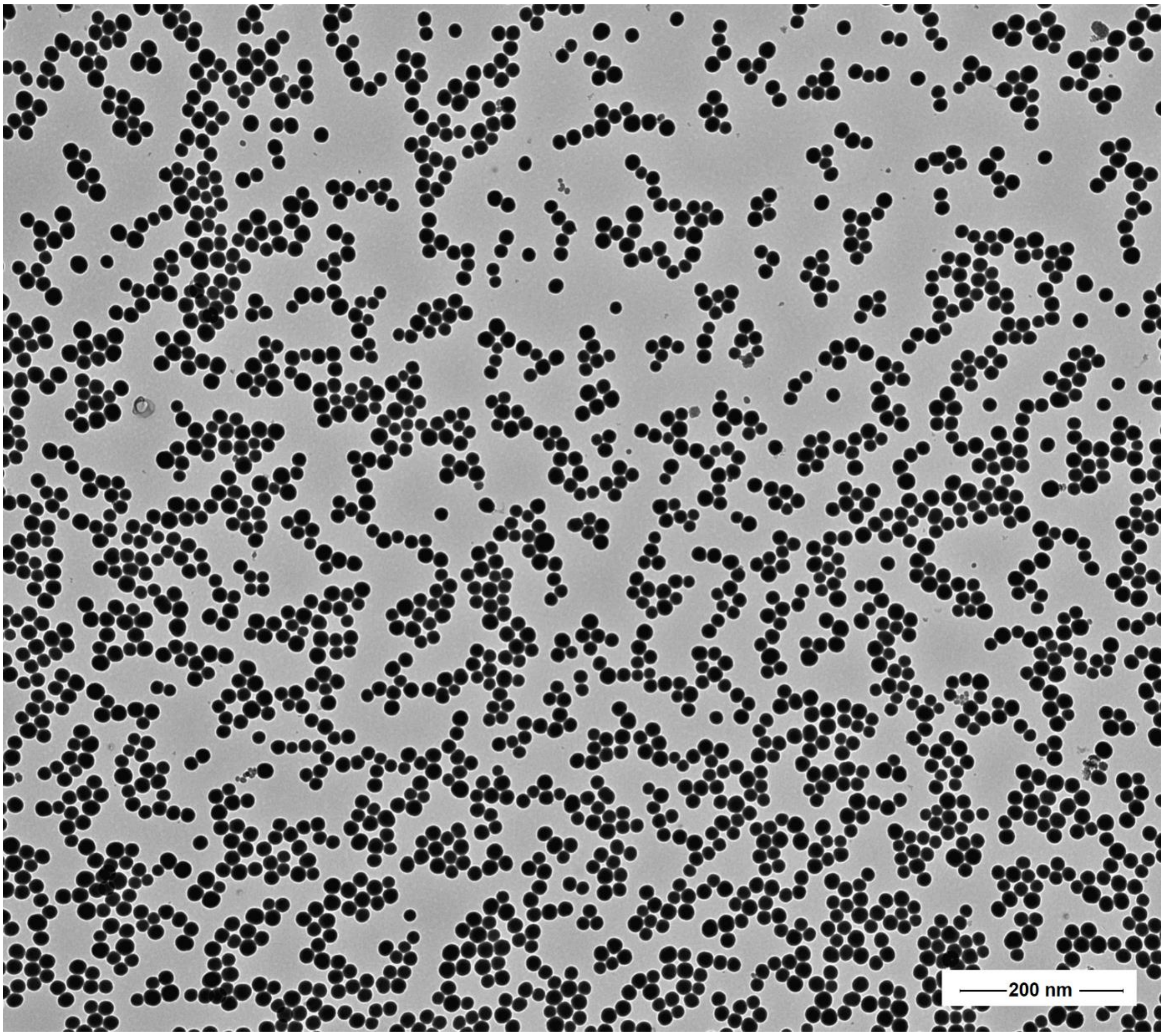

Supplementary Figure S7. TEM images of surface modified silica nanoparticles (SNPs): 100,000× magnification, accelerating voltage: 80 kV; d_{TEM} = 20 nm. PDI = 0.041.

The morphology of hydrophilic silica was examined with transmission electron microscopy (TEM, JEM-1400, JEOL Ltd., Tokyo, Japan). The drop of the sample suspension was drop-cast onto 200 mesh copper grid coated with carbon film (EMR Carbon support grids, Micro to Nano Ltd, Haarlem, The Netherlands) and dried overnight in vacuum desiccators.

Supplementary Table S1. Chemical composition of *Artemisia annua* essential oil. Abbreviation: LRI ref are values reported in FFNSC 3.01 library; LRI exp are experimental values calculated on SLB-5ms and Supelcowax-10 columns; % MS Sim. is database spectral similarity. The content is expressed as relative abundance.

		**SLB-5ms**				**Supelcowax-10**			
**ID**	Compounds	% MS Sim.	LRI ref	LRI exp	Sample Area %	% MS Sim.	LRI ref	LRI exp	Sample Area %
**1**	Hexanal	91	802	801	0.05	92	480	477	0.05
**2**	Hex-(2E)-enal	92	850	850	0.07	92	631	628	0.06
**3**	Artemisia triene	95	922	923	0.40	-	-	-	nd
**4**	α-Thujene	97	926	927	0.05	96	428	427	0.05
**5**	α-Pinene	96	934	933	0.10	95	425	427	0.11
**6**	Camphene	97	951	953	0.02	91	462	459	0.23
**7**	Thuja-2,4(10)-diene	96	955	953	0.06	97	521	519	0.06

**8**	Benzaldehyde	98	963	960	0.05	95	925	931	0.03
**9**	Sabinene	97	974	972	0.12	97	517	518	0.11
**10**	β-Pinene	97	980	978	0.03	93	502	505	0.02
**11**	2,3-Dehydro-1,8-cineol	87	992	991	0.17	95	602	-	0.14
**12**	Yomogi alcohol	97	995	996	1.29	95	810	812	1.32
**13**	α-Phellandrene	92	1008	1007	0.03	89	570	574	0.04
**14**	α-Terpinene	96	1019	1018	0.31	97	589	586	0.28
**15**	*p*-Cymene	95	1026	1025	0.38	97	676	678	0.35
**16**	Santolina alcohol	86	1031	1033	0.07	96	812	814	0.05
**17**	Eucalyptol	98	1033	1032	0.55	96	617	614	0.57
**18**	Artemisia ketone	92	1058	1056	4.43	91	756	754	3.98
**19**	γ-Terpinene	91	1060	1058	*	95	651	654	0.47
**20**	(Z)-Sabinene hydrate	91	1071	1069	0.13	92	964	-	0.21
**21**	Artemisia alcohol	94	1081	1079	1.68	94	914	913	1.74
**22**	Terpinolene	93	1088	1086	0.15	95	686	681	0.13
**23**	*p*-Cymenene	94	1093	1093	0.05	89	846	847	0.02
**24**	(E)-Sabinene hydrate	86	1099	1103	0.15	92	872	873	0.15
**25**	1,3,8-*p*-Menthatriene	-	-	-	nd	88	624	626	0.01
**26**	*n*-Nonanal	86	1106	1107	0.08	91	805	807	0.04
**27**	dehydro-Sabina ketone	89	1122	1122	0.03	-	-	-	nd
**28**	(Z)-, *p*-Menth-2-en-1-ol	95	1127	1124	0.10	85	968	968	0.09
**29**	α-Campholenal	92	1129	1125	0.10	91	898	899	0.09
**30**	(E)-Verbenol	-	-	-	nd	91	1080	1079	0.70
**31**	(E)-Pinocarveol	93	1145	1141	7.55	94	1056	1055	7.60
**32**	Camphor	95	1151	1149	7.06	96	920	918	6.82
**33**	β-Pinene oxide	87	1158	1156	1.25	-	-	-	nd
**34**	Sabina ketone	93	1160	1157	0.16	90	1035	1035	0.13
**35**	Pinocarvone	95	1166	1164	3.22	95	971	971	2.91
**36**	δ-Terpineol	94	1173	1170	0.43	95	1075	1076	0.35*B
**37**	Borneol	96	1175	1173	0.40	94	1102	-	1.71*C
**38**	Terpinen-4-ol	91	1184	1184	1.75	90	1005	1002	1.66
**39**	Myrtenal	95	1186	1196	0.11	87	1030	1034	1.51
**40**	*p*-Cymen-8-ol	91	1189	1189	0.15	91	1247	1243	0.18
**41**	(E)-Isocarveol	85	1191	1189	0.12	90	1193	1192	0.15
**42**	*p*-Mentha-1,5-dien-7-ol	85	1195	1194	0.06	91	1198	1191	0.08
**43**	α-Terpineol	92	1198	1195	2.19	95	1099	1099	1.71*C
**44**	Myrtenol	95	1201	1202	*	96	1188	1191	0.85
**45**	Verbenone	94	1211	1208	0.12	-	-	-	nd
**46**	(E)-Carveol	93	1222	1223	0.39	91	1232	1232	0.40
**47**	hex-(3Z)-enyl, 2-methyl Butanoate	86	1231	1231	0.08	-	-	-	nd
**48**	(Z)-Carveol	86	1236	1232	0.11	90	1262	1262	0.11
**49**	Carvone	94	1247	1246	0.11	92	1135	1133	0.05
**50**	*n*-Tridec-1-ene	95	1293	1292	0.13	95	750	-	0.01
**51**	(E)-Pinocarvyl acetate	92	1298	1296	0.07	85	1051	1052	0.07
**52**	Thymol	87	1293	1293	0.09	-	-	-	nd
**53**	α-Cubebene	90	1345	1350	0.16	98	863	864	0.19
**54**	Eugenol	85	1356	1357	0.42	87	1554	1552	0.61
**55**	Cyclosativene	93	1372	1367	0.02	-	-	-	nd
**56**	α-Copaene	93	1380	1375	2.75	95	896	898	2.87

**57**	β-Cubebene	95	1391	1392	0.32	96	941	942	0.36[*A]
**58**	(Z)-Jasmone	93	1396	1394	0.12	86	1326	1330	0.10
**59**	Ylanga-2,4(15)-diene	89	1410	1411	0.16	-	-	-	nd
**60**	(E)-Caryophyllene	95	1425	1424	5.26	96	996	996	5.55
**61**	β-Copaene	90	1434	1432	0.07	93	942	944	0.36[*A]
**62**	(E)-, β-Farnesene	91	1455	1452	4.80	96	1070	1070	4.07
**63**	Sesquisabinene	-	-	-	nd	92	1166	1170	0.05
**64**	α-Humulene	91	1459	1460	0.34	95	1067	1067	0.40
**65**	Cadina-4,11-diene	88	1462	1458	0.15	-	-	-	nd
**66**	Selina-4,11-diene	91	1478	1476	0.29	86	1074	1076	0.35[*B]
**67**	γ-Muurolene	-	-	-	nd	95	1086	1086	0.13
**68**	Amorpha-4,7(11)-diene	90	1481	1480	0.06	-	-	-	nd
**69**	(E)-, β-Ionone	92	1483	1482	0.08	-	-	-	nd
**70**	Germacrene D	89	1485	1480	0.52	92	1107	-	0.59
**71**	β-Selinene	97	1497	1492	12.27	94	1116	1117	12.75
**72**	δ-Cadinene	94	1523	1518	0.42	93	1152	1152	0.47
**73**	(E)-Calamenene	89	1526	1527	0.12	88	1226	1229	0.05
**74**	α-Calacorene	90	1546	1544	0.10	87	1306	1310	0.12
**75**	(E)-Nerolidol	87	1563	1561	0.15	93	1431	1431	0.15
**76**	Spathulenol	84	1581	1576	1.75	89	1510	1512	1.15
**77**	Caryophyllene oxide	91	1589	1587	8.64	93	1371	1364	8.71
**78**	β-Copaen-4-alfa-ol	-	-	-	nd	87	1532	1534	0.39
**79**	Salvial-4(14)-en-1-one	88	1599	1596	0.62	94	1395	1397	0.57
**80**	Humulene epoxide II	86	1616	1613	0.73	90	1425	1431	0.43
**81**	Eudesma-4(15),11-dien-5-ol	85	1639	-	1.06	-	-	-	nd
**82**	(Z)-Cadin-4en-7-ol	88	1642	1638	*	89	1485	1486	0.40
**83**	Eudesma-4(15),7-dien-1-beta-ol	88	1665	1670	0.75	87	1730	-	1.06
**84**	Mustakone	89	1681	1681	1.27	-	-	-	nd
**85**	Neophytadiene	94	1837	1836	0.33	-	-	-	nd
**86**	Phytone	90	1842	1841	0.25	89	1516	1514	0.54
**87**	Nonadecane	95	1901	1900	0.29	88	1295	1296	0.41
**88**	*n*-Heneicosane	95	2101	2100	0.12	88	1488	1488	0.27
**89**	Phytol	92	2107	2111	0.17	95	1982	1983	0.22
**90**	*n*-Tricosane	96	2301	2300	0.24	94	1681	1678	0.29
	Not identified [a]				18.99				21.43
	**TOTAL**				**100.00**				**100.00**

nd: not detected

*: coelution

*A: coelution between β-Cubebene and β-Copaene on Supelcowax-10 column

*B: coelution between δ-Terpineol and Selina-4,11-diene on Supelcowax-10 column

*C: coelution between α-Terpineol and Borneol on Supelcowax-10 column

[a] : sum of not identified compounds.
